# Dermatol in Gynecology

**Published:** 1892-10

**Authors:** 


DERMATOL IN GYNECOLOGY.—Asch (*Cent. fur Gynecologie; Condensed Extracts*) deems dermatol of comparatively little antiseptic value, but owing to its decided siccative action, he considers it indirectly useful in further aseptic healing of wounds.

He prefers it to iodoform in lacerated cervix after suture, but not in inoperable ichorous carcinomata. In recent ruptured perineum and in perineoplasty, he considers it most desirable, as it protects the wound and sutures from saturation and uncleanliness better than any other remedy. A single application of a thick layer of dermatol, loosely covered with dermatol gauze, will keep the site of operation dry and clean for a several days.

The author reports success in vaginal catarrh from using dermatol tampons.

In an extensive ulcer of the portico vaginalis and of the fornix he obtained rapid healing with dermatol.

In intertrigo, one or two applications of a thick layer of dermatol sufficed to produce marked improvement.

Dermatol is recommended as a protective dressing for the healthy skin, when using irritant substances upon a diseased point.—*Annals of Gynecology.*

